# Uncoupling Protein-1 Modulates Anxiety-Like Behavior in a Temperature-Dependent Manner

**DOI:** 10.1523/JNEUROSCI.2509-21.2022

**Published:** 2022-10-05

**Authors:** Spyridon Sideromenos, Anna Gundacker, Maria Nikou, Raimund Oberle, Orsolya Horvath, Peter Stoehrmann, Timo Partonen, Daniela D. Pollak

**Affiliations:** ^1^Department of Neurophysiology and Neuropharmacology, Center for Physiology and Pharmacology, Medical University of Vienna, 1090 Vienna, Austria; ^2^Institute of Medical Chemistry and Pathobiochemistry, Center for Pathobiochemistry and Genetics, Medical University of Vienna, 1090 Vienna, Austria; ^3^Mental Health Unit, Finnish Institute for Health and Welfare, FI-00271 Helsinki, Finland

**Keywords:** anxiety, brown adipose tissue, fear, mouse behavior, uncoupling protein 1

## Abstract

A strong bidirectional link between metabolic and psychiatric disorders exists; yet, the molecular basis underlying this interaction remains unresolved. Here we explored the role of the brown adipose tissue (BAT) as modulatory interface, focusing on the involvement of uncoupling protein 1 (UCP-1), a key metabolic regulator highly expressed in BAT, in the control of emotional behavior. Male and female constitutive UCP-1 knock-out (KO) mice were used to investigate the consequences of UCP-1 deficiency on anxiety-related and depression-related behaviors under mild thermogenic (23°C) and thermoneutral (29°C) conditions. UCP-1 KO mice displayed a selective enhancement of anxiety-related behavior exclusively under thermogenic conditions, but not at thermoneutrality. Neural and endocrine stress mediators were not affected in UCP-1 KO mice, which showed an activation of the integrated stress response alongside enhanced fibroblast-growth factor-21 (FGF-21) levels. However, viral-mediated overexpression of FGF-21 did not phenocopy the behavioral alterations of UCP-1 KO mice and blocking FGF-21 activity did not rescue the anxiogenic phenotype of UCP-1 KO mice. No effects of surgical removal of the intrascapular BAT on anxiety-like behavior or FGF-21 levels were observed in either UCP-1 KO or WT mice. We provide evidence for a novel role of UCP-1 in the regulation of emotions that manifests as inhibitory constraint on anxiety-related behavior, exclusively under thermogenic conditions. We propose this function of UCP-1 to be independent of its activity in the BAT and likely mediated through a central role of UCP-1 in brain regions with converging involvement in energy and emotional control.

**SIGNIFICANCE STATEMENT** In this first description of a temperature-dependent phenotype of emotional behavior, we propose uncoupling protein-1 (UCP-1), the key component of the thermogenic function of the brown adipose tissue, as molecular break controlling anxiety-related behavior in mice. We suggest the involvement of UCP-1 in fear regulation to be mediated through its expression in brain regions with converging roles in energy and emotional control. These data are important and relevant in light of the largely unexplored bidirectional link between metabolic and psychiatric disorders, which has the potential for providing insight into novel therapeutic strategies for the management of both conditions.

## Introduction

Mental disorders are often associated with somatic comorbidities, which prominently include metabolic disorders (e.g., metabolic syndrome, type II diabetes mellitus; Nousen et al., 2013). At the same time, metabolic disturbances are frequently paralleled by psychiatric manifestations with relevance to anxiety and depressive disorders (Kahl et al., 2015). While the mechanistic basis for the bidirectional association between metabolic and psychiatric disorders (Fulton et al., 2022) has not been defined, shared pathophysiological principles may include common neural circuits (Cai et al., 2014; Daviu et al., 2019; Bruschetta et al., 2020; Xia et al., 2021) and endocrine mediators [e.g., glucocorticoids (Moraitis et al., 2017), leptin (Ates et al., 2014), insulin (Milstein and Ferris, 2021), and adiponectin (Sun et al., 2019)].

A part of the well known key players relevant to the regulation of metabolic processes, the brown adipose tissue (BAT) has emerged as an important metabolic organ within the last decades (Cypess et al., 2009; van Marken Lichtenbelt et al., 2009; Virtanen et al., 2009). BAT is a thermogenic structure that is activated upon cold exposure and dissipates energy as heat through a process termed nonshivering thermogenesis, which is mediated by uncoupling protein-1 (UCP-1; Cannon and Nedergaard, 2004). UCP-1 is located in the inner mitochondrial membrane of brown and beige adipocytes, where it acts to uncouple chemical energy (Cannon and Nedergaard, 2004). Related to its role to increase energy expenditure, BAT mass and activity are inversely correlated with obesity and body mass index (Cypess et al., 2009) and certain UCP-1 polymorphisms are associated with obesity and obesity-related conditions (Jia et al., 2010; Chathoth et al., 2018).

In addition to its role in thermogenesis, BAT is also a secretory organ that releases a variety of adipokines (batokines) with autocrine/paracrine functions and regulatory effects on distant organs and tissues, which also contribute to the beneficial metabolic consequences of BAT activation (Villarroya et al., 2017). Fibroblast growth factor 21 (FGF-21) is one of the most intensively studied batokines (Hondares et al., 2011; Keipert et al., 2015), and several of the metabolically favorable effects of BAT activation are also attributed to FGF-21 (Hondares et al., 2011). Some functions of FGF-21 are further mediated through its central effects, where the expression of β-clotho, the obligatory coreceptor to FGF receptors, defines its sites of action (Charmandari et al., 2005; Bookout et al., 2013).

Also UCP-1, which originally was thought to be restricted to adipose tissues, is found in the brain, with significant expression in the ventromedial hypothalamus (VMH) and the amygdala (Claflin et al., 2022), regions involved in both temperature and emotional control processes (Charmandari et al., 2005; Morrison, 2016; Soto et al., 2019).

Psychiatric and metabolic disorders are not only severe disease conditions, which significantly affect the quality of life of the affected individuals but are also of critical public health and socioeconomic relevance (Wittchen et al., 2011). Against this background, the lack of information on the conceptual basis of their high degree of comorbidity is worrying. We here approached this problem by testing the hypothesis that reduced BAT activity could be involved in the pathogenesis of emotional disorders, focusing on the role of UCP-1 as a possible molecular mediator in the pathophysiological interaction between metabolic and emotional disturbances.

## Materials and Methods

### Animals

Heterozygous UCP-1 mice were purchased from The Jackson Laboratory (stock #003124) and used for breeding of UCP-1 knock-out (KO) mice. Male and female wild-type (WT) and UCP-1 KO littermates were used for all experiments on UCP-1 deficiency. Breeding occurred at regular housing temperature (23°C), and offspring were weaned at postnatal day 21. After weaning, mice were housed with same-sex littermates either at regular housing temperature (23°C) or at thermoneutrality (29°C) using an environmental chamber (HPP750life, Memmert) for temperature control. All mice were maintained at a regular 12 h light/dark cycle (lights on at 8:00 A.M.) and food (regular chow diet) and water available were *ad libitum* unless indicated otherwise. For FGF-21 overexpression experiments, C5BL6/N mice were purchased from Charles River. All animal experiments were conducted in agreement with the ARRIVE (Animal Research: Reporting of In Vivo Experiments) guidelines and the U.K. Animals (Scientific Procedures Act, 1986) and associated guidelines (EU Directive 2010/63/EU for animal experiments) and were approved by the national ethical committee on animal care and use (Bundesministerium für Wissenschaft und Forschung: BMBWF-66.009/0175-V/3b/2019).

### Genotyping

Ear punches were collected from 3-week-old mice. DNA was extracted from the biopsy specimens using a DNA extraction kit (BioCat). For the PCR-based amplification the following primers were used: WT forward, TCGTCATCAATAAGGGGAAAC; WT reverse, CTTCTTCCCTGATGCTCCAT; KO forward, GATCCCCCGGGCAATTCT; and KO reverse, CTTCCTGACTAGGGGAGGAGT. Electrophoresis was conducted in a 2% agarose gel for 1.5 h at 150 V. Samples from KO mice present with a single band at 206 bp, from WT mice a single band at 279 bp, and from heterozygous mice two bands at 206 and 279 bp.

### Behavioral experiments

All mice were singled housed before behavioral testing, since group-housed mice tend to huddle together, which could change the thermal environment, thus possibly biasing the thermoregulatory requirement of the experimental design.

For the baseline characterization of UCP-1 KO mice housed under thermogenic conditions (23°C), we used three different cohorts of mice. In the first cohort of mice, the behavioral consequences of UCP-1 KO mice were evaluated in the elevated plus maze (EPM), light-dark box (LD-BOX), forced swim test (FST), novelty-suppressed feeding (NSF) test, and open field test (OFT). The second cohort of mice was tested in the fear-conditioning paradigm. To exclude motor impairment as a confounding factor, a third cohort of WT and UCP-1 KO littermates was tested in the rotarod (RR) test. For the baseline characterization of UCP-1 KO mice under thermoneutral conditions (29°C), two different cohorts of mice were used. In the first cohort, the behavioral consequences of UCP-1 KO mice were evaluated in the EPM, LD-BOX, FST, NSF, and OFT. The second cohort of mice was tested in the contextual fear-conditioning paradigm. For all other behavioral experiments, a single cohort of mice was subjected to all behavioral tests.

#### Elevated plus maze.

The EPM test was performed as previously described with the EPM consisting of two open and two closed arms (Dorninger et al., 2019). Mice were always placed at the center of the maze, facing the open arms. The intensity of the light was set at 40 lux for the open arms and 10 lux for the closed arms. Mice were placed for 5 min in the EPM, and the behavior was tracked automatically (VIDEOTRACK, Viewpoint). The percentage of open arm entries (open arm entries/total arm entries *100) was calculated to evaluate anxiety-like behavior (Pellow et al., 1985).

#### Light-dark box.

The LD-BOX consisted of a rectangular arena (27.3 × 27.3 cm^2^) that was divided with an insert into two equal compartments. One compartment of the arena was brightly illuminated (250 lux), while the other one was dark (maximum, 5 lux). Behavior was recorded with an automated system (Activity Monitor; catalog #SOF 811, Med Associates), and the time spent in the light compartment was determined and used to indicate anxiety-like behavior (Belzung and Griebel, 2001).

#### Novelty-suppressed feeding.

NSF was performed according to a previously published procedure (Reisinger et al., 2019). In brief, mice were fasted for 24 h, and body weight loss as a result of food deprivation was determined. Mice that lost >20% of their initial body weight were excluded from the study. On the day of testing, a food pellet was fixed on a paper and placed in the center of a brightly illuminated arena (800 lux), filled with bedding. Mice were always placed in the corner of the arena and the latency to start eating the food pellet was recorded and considered as an indicator for anxiety-like behavior. The maximum duration was 15 min. After the termination of the test, mice were transferred back to their home cage, where they were given access to a single food pellet for 5 min, and homecage food consumption was recorded to control for possible unspecific changes in appetitive behavior.

#### Forced swim test.

The FST was conducted using an automated movement-tracking software (VideoTrack version 3, Viewpoint) as reported previously (Reisinger et al., 2020b). The test lasted for 6 min, and the percentage of immobility during the last 4 min of the test was used as an indicator of despair-like behavior related to depression.

#### Open field test.

Mice were placed in a rectangular arena (27.3 × 27.3 cm^2^; 300 lux). Locomotor behavior was recorded for 30 min with an automated system (Activity Monitor, Med Associates), and the total ambulatory distance was calculated (Gabriel et al., 2020).

#### Rotarod.

The RR test was used to assess motor coordination and was conducted as previously described (Muratspahić et al., 2021). The mice were placed on a rotating drum with the speed gradually increasing from 4 to 40 rounds/min. Every mouse was subjected to the RR test three times. The intertrial interval was set to 30 min. The latency to fall from the rotating drum was automatically recorded (Med Associates). The average latency to fall in the three trials was calculated and used as an index of motor coordination.

#### Fear conditioning.

An automated video-based recording and conditioning system was used (Med Associates) applying a standard protocol (Dorninger et al., 2019). Briefly, mice were trained for 2 consecutive days. Each training session consisted of two pairings of mild footshock [0.60 mA; unconditional stimulus (US)] and white noise [75 dB; conditional stimulus (CS)]. Contextual fear conditioning was tested 24 h after the last training day by placing the mice in the same chamber without US or CS presentation for a period of 5 min. The percentage of time spent immobile was quantified using the near-infrared Video Conditioning System for recording (Med Associates) and Video Freeze software (Med Associates) for analysis

### Surgical removal of brown adipose tissue

Anesthesia was induced with 5% isoflurane and maintained with 2.5% isoflurane (Forane, AbbVie). The interscapular BAT (iBAT) was surgically removed according to a published procedure (Connolly et al., 1982). A small incision was made along the dorsal midline. iBAT was exposed and carefully removed. For sham-operated mice, iBAT was exposed but not removed. The health status of all animals was closely monitored after the surgical procedure, and the body weight was measured. None of the mice lost >20% of the initial body weight or showed apparent signs of discomfort.

### Serum analytes (corticosterone, epinephrine, norepinephrine, and FGF-21)

To evaluate the circadian rhythmicity of corticosterone (CORT) levels, blood was collected at four time points [circadian time (CT): CT4, CT10, CT16, CT22]. Blood sampling during the dark phase of the mice was performed under dim red light. To measure stress-induced CORT levels, mice were restrained in a modified 50 ml Falcon tube for 15 min (Zalutskaya et al., 2007). Mice were deeply anaesthetized with isoflurane, and the trunk blood was collected either immediately (time point 0) or 30 min after (time point 30) the termination of the stress exposure. Blood samples were kept at room temperature for at least 45 min before centrifugation at 1200 × *g* for 10 min for serum collection. Serum corticosterone (catalog #ADI-900–097, Enzo Life Sciences), epinephrine (Epi; catalog #E-EL-0045, Elabscience), norepinephrine (nor-Epi; catalog #E-EL-0047, Elabscience), and FGF-21 (catalog #MF2100, R&D Systems) were measured using commercially available enzyme immunoassay kits following manufacturer instruction.

### FGF-21 overexpression

The production of liver-targeting, FGF-21-expressing adeno-associated virus (AAV) particles of the serotype AAV2.8 was performed as previously described in detail (Xiao et al., 1998; Gray et al., 2011; Körbelin et al., 2016). Briefly, the full-length murine FGF-21 cDNA was inserted into a pAAV-MCS plasmid-containing AAV inverted terminal repeat using BstBI (forward) and BsrGI (reverse) restriction enzyme sites. Together with a pAAV rep2 cap8 transfer plasmid and an AdpXX6 helper plasmid, HEK cells were cotransfected, and virus particles purified from cell pellets and supernatants using iodixanol density gradients (Xiao et al., 1998; Gray et al., 2011; Körbelin et al., 2016). A dose of 1 × 10^10^ viral genome copies (vgc) was used for all additional experiments, similar to previous studies (Jimenez et al., 2018). AAV particles were diluted with PBS (Thermo Fisher Scientific) and 200 µl of a PBS solution containing 1 × 10^10^ vgc were injected intravenously into mice. Control mice received a tail vein injection of the same volume and number of vgc of AAV particles without transgene.

### FGF-21 inhibition

Polyclonal antibodies against mouse FGF-21 (catalog #12180, Immunodiagnostics) were administered intraperitoneally at a dose of 250 µg/kg according to a published procedure (Li et al., 2020) 6 h before behavioral testing (Liu et al., 2019). The effect of FGF-21 inhibition in UCP-1 KO was evaluated in the contextual fear test. Control UCP-1 KO mice received a same dose of IgG (catalog #ab18469, Abcam).

### Gene expression analysis

Animals were killed by cervical dislocation, and tissue samples were collected, rapidly frozen in liquid nitrogen, and stored at −80°C, until further processing. RNA was extracted using the miRNeasy Mini Kit (catalog #74104, Qiagen) following the manufacturer instructions. After RNA isolation, genomic DNA was removed using the DNA-free kit (catalog #AM1906, Thermo Fisher Scientific). The concentration and purity of RNA were determined using a nanodrop photometer (NanoPhotometer 7122 version 2.3.1, IMPLEN). At least 150 ng of RNA were transcribed into cDNA using the RevertAid First Strand cDNA Synthesis Kit (catalog #K1621, Thermo Fisher Scientific) following the instructions of the manufacturer. Relative levels of the selected transcripts were measured by quantitative real-time PCR (qRT-PCR) using the Go-Taq qPCR Master Mix 2× (catalog #A6002, Promega) and a CFX Connect PCR cycler (BIO-RAD). Relative differences in gene expression were calculated according to the 2^-ΔΔCt^ method (Schmittgen and Livak, 2008). β-Actin was used as an internal housekeeping gene for brain samples, and 36B4 for adipose tissue samples and liver. A list with all primers sequences is provided in Extended Data Figure 3-1.

### Experimental design and statistical analyses

All analyses were performed by an investigator blinded to the experimental condition of the animals. *N* numbers, full statistics, and ρ values are reported for each main effect, and all interactions are listed where relevant in the main text; a complete report including sample sizes for each experiment is given Table 1. Sample sizes were determined according to our own experience, and data provided in the literature (Kong et al., 2015; Dorninger et al., 2019; Reisinger et al., 2019, 2020a,b; Berger et al., 2020; Gabriel et al., 2020). All statistical analyses were conducted using GraphPad Prism 7. Data were tested for normality using the Kolmogorov–Smirnov test before further statistical evaluation. Outliers were removed using the Tukey's boxplot method. For all analyses, *p* < 0.05 was considered statistically significant.

**Table 1.** Full statistical reporting

Figures	Experiment	Parameter	Statistical test	*n*/group	Factor	Statistics, df	*p*	Fisher's (uncorrected) multiple-comparisons test
1 *A*	Light-dark box	Time in light Box (s)	Two-way ANOVA		Interaction	*F*_(1,40)_ = 1.141	*p* = 0.2918	**WT males vs KO males; *p* = 0.0012**
				10–14	Sex	*F*_(1,0)_ = 1.801	*p* = 0.1872	WT females vs KO females; *p* = 0.09
					**Genotype**	***F*_(1,40)_ = 13.21**	***p* = 0.0008**	
1 *B*	Novelty-suppressed feeding	Latency to feed (s)	Two-way ANOVA		Interaction	*F*_(1,40)_ = 0.191	*p* = 0.6645	**WT males vs KO males; *p* = 0.0057**
				10–14	Sex	*F*_(1,40)_ = 2.026	*p* = 0.1624	**WT females vs KO females; *p* = 0.0033**
					**Genotype**	***F*_(1,40)_ = 18.29**	***p* = 0.0001**	
1 *B'*	5 min food intake after NSF	Food intake (g)	Two-way ANOVA		Interaction	*F*_(1,42)_ = 1.423	*p* = 0.2396	
				10–14	Sex	*F*_(1,42)_ = 0.6397	*p* = 0.4283	
					Genotype	*F*_(1,42)_ = 0.6796	*p* = 0.4144	
1 *C*	Elevated plus maze	Open arm entries (%)	Two-way ANOVA		Interaction	*F*_(1,42)_ = 0.2757	*p* = 0.6023	WT males vs KO males; *p* = 0.0628
				10–14	Sex	*F*_(1,42)_ = 0.8395	*p* = 0.3648	**WT females vs KO females; *p* = 0.0219**
					**Genotype**	***F*_(1,42)_ = 9.304**	***p* = 0.0039**	
1 *D*	Contextual fear	Freezing (%)	Two-way ANOVA		Interaction	*F*_(1,37)_ = 1.215	*p* = 0.2774	**WT males vs KO males; *p* = 0.0083**
				8–14	Sex	*F*_(1,37)_ = 3.628	*p* = 0.0646	WT females vs KO females; *p* = 0.0942
					**Genotype**	***F*_(1,37)_ = 10.56**	***p* = 0.0025**	
1 *E*	FST	Immobility (%)	Two-way ANOVA	11	Interaction	*F*_(1,42)_ = 0.02625	*p* = 0.8721	WT males vs WT females; *p* = 0.076
					**Sex**	***F*_(1,42)_ = 5.751**	***p* = 0.0210**	KO males vs KO females; *p* = 0.1247
					Genotype	*F*_(1,42)_ = 1.928	*p* = 0.1723	
1 *F*	Open field test	Ambulatory distance (m)	Two-way ANOVA	10–14	Interaction	*F*_(1,41)_ = 0.417	*p* = 0.5220	Not applicable
					Sex	*F*_(1,41)_ = 0.001236	*p* = 0.9721	
					Genotype	*F*_(1,41)_ = 0.3191	*p* = 0.5752	
1 *G*	Rotarod	Latency to fall (s)	Two-way ANOVA	5–6	Interaction	*F*_(1,19)_ = 2.33	*p* = 0.1434	Not applicable
					Sex	*F*_(1,19)_ = 0.1748	*p* = 0.6805	
					Genotype	*F*_(1,19)_ = 0.8643	*p* = 0.3642	
2 *A*	Light-dark box	Time in light Box (s)	Two-way ANOVA		Interaction	*F*_(1,40)_ = 2.689	*p* = 0.1089	Not applicable
				8–13	Sex	*F*_(1,40)_ = 2.219	*p* = 0.1442	
					Genotype	*F*_(1,40)_ = 0.3824	*p* = 0.5398	
2 *B*	Novelty-suppressed feeding	Latency to feed (s)	Two-way ANOVA		Interaction	*F*_(1,39)_ = 1.302	*p* = 0.2609	Not applicable
				9–13	Sex	*F*_(1,39)_ = 0.03546	*p* = 0.8516	
					Genotype	*F*_(1,39)_ = 2.447	*p* = 0.1258	
2 *B'*	5 min food intake after NSF	Food intake (g)	Two-way ANOVA		Interaction	*F*_(1,39)_ = 0.4558	*p* = 0.5036	Not applicable
				9–13	Sex	*F*_(1,39)_ = 0.1679	*p* = 0.6842	
					Genotype	*F*_(1,39)_ = 0.04321	*p* = 0.8364	
2 *C*	Elevated plus maze	Open arm entries (%)	Two-way ANOVA		Interaction	*F*_(1,42)_ = 0.8859	*p* = 0.3520	Not applicable
				8–14	Sex	*F*_(1,42)_ = 0.9844	*p* = 0.3268	
					Genotype	*F*_(1,42)_ = 2.717	*p* = 0.1067	
2 *D*	Contextual fear	Freezing (%)	Two-way ANOVA		Genotype	*F*_(1,40)_ = 0.1525	*p* = 0.6983	Not applicable
				10–12	Sex	*F*_(1,40)_ = 0.001269	*p* = 0.9718	
					Genotype X Sex	*F*_(1,40)_ = 0.7426	*p* = 0.3940	
2 *E*	FST	Immobility (%)	Two-way ANOVA	9–14	Interaction	*F*_(1,42)_ = 0.008293	*p* = 0.9279	Not applicable
					Sex	*F*_(1,42)_ = 0.7225	*p* = 0.4002	
					Genotype	*F*_(1,42)_ = 1.921	*p* = 0.1731	
2 *F*	Open field test	Ambulatory distance (m)	Two-way ANOVA	9–14	Interaction	*F*_(1,42)_ = 1.258	*p* = 0.2683	Not applicable
					Sex	*F*_(1,42)_ = 0.522	*p* = 0.4740	
					Genotype	*F*_(1,42)_ = 1.2	*p* = 0.2795	
3 *A*	Serum norepinephrine	ng/ml	Two-way ANOVA		Interaction	*F*_(1,21)_ = 0.04829	*p* = 0.8282	Not applicable
				5–7	Sex	*F*_(1,21)_ = 0.007154	*p* = 0.9334	
					Genotype	*F*_(1,21)_ = 0.1336	*p* = 0.7184	
3 *B*	Serum epinephrine	ng/ml	Two-way ANOVA		Interaction	*F*_(1,19)_ = 0.7293	*p* = 0.4037	Not applicable
				5–7	Sex	*F*_(1,19)_ = 0.9197	*p* = 0.3496	
					Genotype	*F*_(1,19)_ = 0.1166	*p* = 0.7365	
3 *C*	Circadian corticosterone	ng/ml	Repeated-measures two-way ANOVA	7–9	Interaction	*F*_(3,56)_ = 0.09773	*p* = 0.9610	**WT: CT 4 vs CT 10, *p* < 0.0001**
					**Circadian time**	***F*_(3,56)_ = 31.16**	***p* < 0.0001**	**WT: CT 10 vs CT 16, *p* < 0.0001**
					Genotype	*F*_(1,56)_ = 4.289e-005	*p* = 0.9948	**WT: CT 10 vs CT 22, *p* < 0.0001**
								**KO: CT 4 vs CT 10, *p* < 0.0001**
								**KO: CT 10 vs CT 16, *p* = 0.0001**
								**KO: CT 10 vs CT 22, *p* < 0.0001**
3 *D*	Stress-induced corticosterone	ng/ml	Two-way ANOVA		Interaction	*F*_(1,15)_ = 0.04102	*p* = 0.8422	**WT: 0 vs 30 min; *p* = 0.0028**
				4–5	**Time after stress**	***F*_(1,15)_ = 24.88**	***p* = 0.0002**	**KO: 0 vs 30 min; *p* = 0.0033**
					Genotype	*F*_(1,15)_ = 1.034	*p* = 0.3253	
3 *E*	ATF-4 expression in iBAT	Relative ATF-4 expression to 36B4	Student's *t* test					Not applicable
				9	**Genotype**	*t* **= 4.596. df = 16**	***p* = 0.0003**	
3 *F*	CHOP-10 expression in iBAT	Relative CHOP-10 expression to 36B4	Student's *t* test					Not applicable
				8–9	**Genotype**	*t* **= 7.359. df = 15**	***p* < 0.0001**	
3 *G*	FGF-21 expression in iBAT	Relative FGF-21 expression to 36B4	Student's *t* test					Not applicable
				8–9	**Genotype**	*t* **= 6.538, df = 15**	***p* < 0.0001**	
3 *H*	Serum FGF-21	pg/ml	Two-way ANOVA		Interaction	*F*_(1,12)_ = 0.2896	*p* = 0.6003	**WT males vs KO males; *p* = 0.0368**
				3–5	**Sex**	***F*_(1,12)_ = 5.101**	***p* = 0.0433**	WT females vs KO females; *p* = 0.2028
					**Genotype**	***F*_(1,12)_ = 6.567**	***p* = 0.0249**	WT males vs WT females; *p* = 0.2746
								KO males vs KO females; *p* = 0.0556
3 *I*	ATF-4 expression in iBAT at thermoneutrality	Relative ATF-4 expression to 36B4	Student's *t* test					Not applicable
				4	Genotype	*t* = 2.089, df = 6	*p* = 0.08	
3 *J*	CHOP-10 expression in iBAT at thermoneutrality	Relative CHOP-10 expression to 36B4	Student's *t* test					Not applicable
				4	Genotype	*t* = 0.4031, df = 6	*p* = 0.7	
3 *K*	FGF-21 expression in iBAT at thermoneutrality	Relative FGF-21 expression to 36B4	Student's *t* test					Not applicable
				3	Genotype	*t* = 1.690, df = 14	*p* = 0.17	
4 *A*	Body weight	Body weight change (%)	Student's *t* test					Not applicable
				9	FGF-21 treatment	*t* **= 6.11, df = 16**	***p* < 0.0001**	
4 *B*	Food intake	Food intake (g) during 3rd week	Student's *t* test					Not applicable
				7–9	FGF-21 treatment	*t* **= 6.517, df = 14**	***p* < 0.0001**	
4 *C*	Water intake	Water intake during 3rd week (ml)	Student's *t* test					Not applicable
				8–9	FGF-21 treatment	*t* **= 6.393, df = 15**	***p* < 0.0001**	
4 *D*	Light-dark box	Time in light Box (s)	Student's *t* test					Not applicable
				8–9	FGF-21 treatment	*t* = 1.899, df = 15	*p* = 0.077	
4 *E*	Elevated plus maze	Open arm entries (%)	Student's *t* test					Not applicable
				8–9	FGF-21 treatment	*t* = 0.1832, df = 15	*p* = 0.8571	
4 *F*	FST	Immobility (%)	Student's *t* test					Not applicable
				9	FGF-21 treatment	*t* = 0.1502, df = 16	*p* = 0.8825	
4 *G*	Contextual fear	Freezing (%)	Student's *t* test					Not applicable
				9	FGF-21 treatment	*t* = 0.8401, df = 16	*p* = 0.4132	
4 *H*	Open field test	Ambulatory distance (m)	Student's *t* test					Not applicable
				8–9	FGF-21 treatment	*t* = 1.288, df = 15	*p* = 0.2173	
4 *I*	Contextual fear	Freezing (%)	Student's *t* test					Not applicable
				5	FGF-21 inhibition	*t* = 1.200, df = 8	*p* = 0.2645	
5 *A*	Elevated plus maze	Open arm entries (%)	Two-way ANOVA		Interaction	*F*_(1,21)_ = 0.2728	*p* = 0.6069	WT sham vs KO sham, *p* = 0.2260
				5–7	iBATx	*F*_(1,21)_ = 0.1309	*p* = 0.7211	WT iBATx vs KO iBATx, *p* = 0.0705
					**Genotype**	***F*_(1,21)_ = 5.021**	***p* = 0.0360**	
5 *B*	Contextual fear	Freezing (%)	Two-way ANOVA		Interaction	*F*_(1,21)_ = 0.5504	*p* = 0.4664	WT sham vs KO sham, *p* = 0.1122
				5–7	iBATx	*F*_(1,21)_ = 2.563	*p* = 0.1244	**WT iBATx vs KO iBATx, *p* = 0.0168**
					**Genotype**	***F*_(1,21)_ = 9.154**	***p* = 0.0064**	
5 *C*	FST	Immobility (%)	Two-way ANOVA		Interaction	*F*_(1,21)_ = 0.3483	*p* = 0.5614	Not applicable
				5–7	iBATx	*F*_(1,21)_ = 2.834	*p* = 0.1071	
					Genotype	*F*_(1,21)_ = 0.5916	*p* = 0.4503	
5 *D*	Open field test	Ambulatory distance (m)	Two-way ANOVA		Interaction	*F*_(1,19)_ = 0.02145	*p* = 0.8851	Not applicable
				5–7	iBATx	*F*_(1,19)_ = 1.015	*p* = 0.3263	
					Genotype	*F*_(1,19)_ = 0.8531	*p* = 0.3673	
5 *E*	Serum FGF-21	pg/ml	Two-way ANOVA		Interaction	*F*_(1,21)_ = 0.3088	*p* = 0.5843	**WT sham vs KO sham, *p* = 0.0367**
				5–7	iBATx	*F*_(1,21)_ = 0.1673	*p* = 0.6866	**WT iBATx vs KO iBATx, *p* = 0.0088**
					**Genotype**	***F*_(1,21)_ = 613.18**	***p* = 0.0016**	
1–1 *A*	UCP-1 expression in hypothalamus in WT and UCP-1 KO mice	ΔCt value						
1–1 *B*	Body weight at regular housing temperature (23°C)	Body weight (g)	Student's *t* test					Not applicable
				12–14	Males	*t* = 0.3545, df = 24	*p* = 0.7261	
				10	Females	*t* = 1.158, df = 18	*p* = 0.262	
2–1	Body weight at thermoneutrality (29°C)	Body weight (g)	Student's *t* test					Not applicable
				9–12	Males	*t* = 0.1998, df = 19	*p* = 0.8438	
				9–11	Females	*t* = 1.25, df = 18	*p* = 0.2274	
3–1 *A*	Serum FGF21 at 9 and 18 d after AAV-FGF21 encoding virus injection	pg/ml	One-way ANOVA					Ctrl vs FGF-21 OE 9 Days, *p* < 0.001
				2	FGF-21 overexpression	***F*_(2,3)_ = 1664**	***p* < 0.001**	Ctrl vs FGF-21 OE 18 Days, *p* < 0.001
3–1 *B*	Liver FGF21 expression 18 d after AAV-FGF21 encoding virus injection	Relative expression to 36B4	Student's *t* test					Not applicable
				3	FGF-21 overexpression	*t* **= 3.612, df = 4**	***p* = 0.0225**	
3–2*A*	Body weight	Body weight change (%)	Student's *t* test					Not applicable
				6	FGF-21 treatment	*t* = 0.6703, df = 10	*p* = 0.5178	
3–2*B*	Food intake	Food intake (g) during 3rd week	Student's *t* test					Not applicable
				6	FGF-21 treatment	***t* = 63.541, df = 10**	***p* = 0.0063**	
3–2*C*	Water intake	Water intake during 3rd week (ml)	Student's *t* test					Not applicable
				6	FGF-21 treatment	***t* = 2.804, df = 10**	***p* = 0.0187**	
3–2*D*	Light-dark box	Time in light Box (s)	Student's *t* test					Not applicable
				9–10	FGF-21 treatment	*t* = 1.559, df = 17	*p* = 0.1374	
3–2*E*	Elevated plus maze	Open arm entries (%)	Student's *t* test					Not applicable
				8–10	FGF-21 treatment	*t* = 0.8984, df = 16	*p* = 0.3823	
3–2*F*	Contextual fear	Freezing (%)	Student's *t* test					Not applicable
				9–10	FGF-21 treatment	*t* = 1160, df = 17	*p* = 0.2616	
3–2*G*	FST	Immobility (%)	Student's *t* test					Not applicable
				9	FGF-21 treatment	*t* = 0.7564, df = 16	*p* = 0.4604	
3–2*H*	Open field test	Ambulatory distance (m)	Student's *t* test	8–10	FGF-21 treatment	*t* = 1.288, df = 15	*p* = 0.2173	Not applicable

Statistically significant results are highlighted in bold.

## Results

### UCP-1 deficiency enhances anxiety-like behavior under thermogenic conditions

To evaluate the consequences of UCP-1 deficiency on emotionality, we applied a series of paradigms for the examination of anxiety-related and depression-related behavior in UCP-1 KO mice and WT littermate controls, after confirming the absence of the UCP-1 transcript in KO mice (Extended Data Fig. 1-1*A*) Consistent with previous reports (Liu et al., 2003), we also found no alterations in the body weight of UCP-1 KO mice at 3 months of age (Extended Data Fig. 1-1*B*).

**Figure 1.**
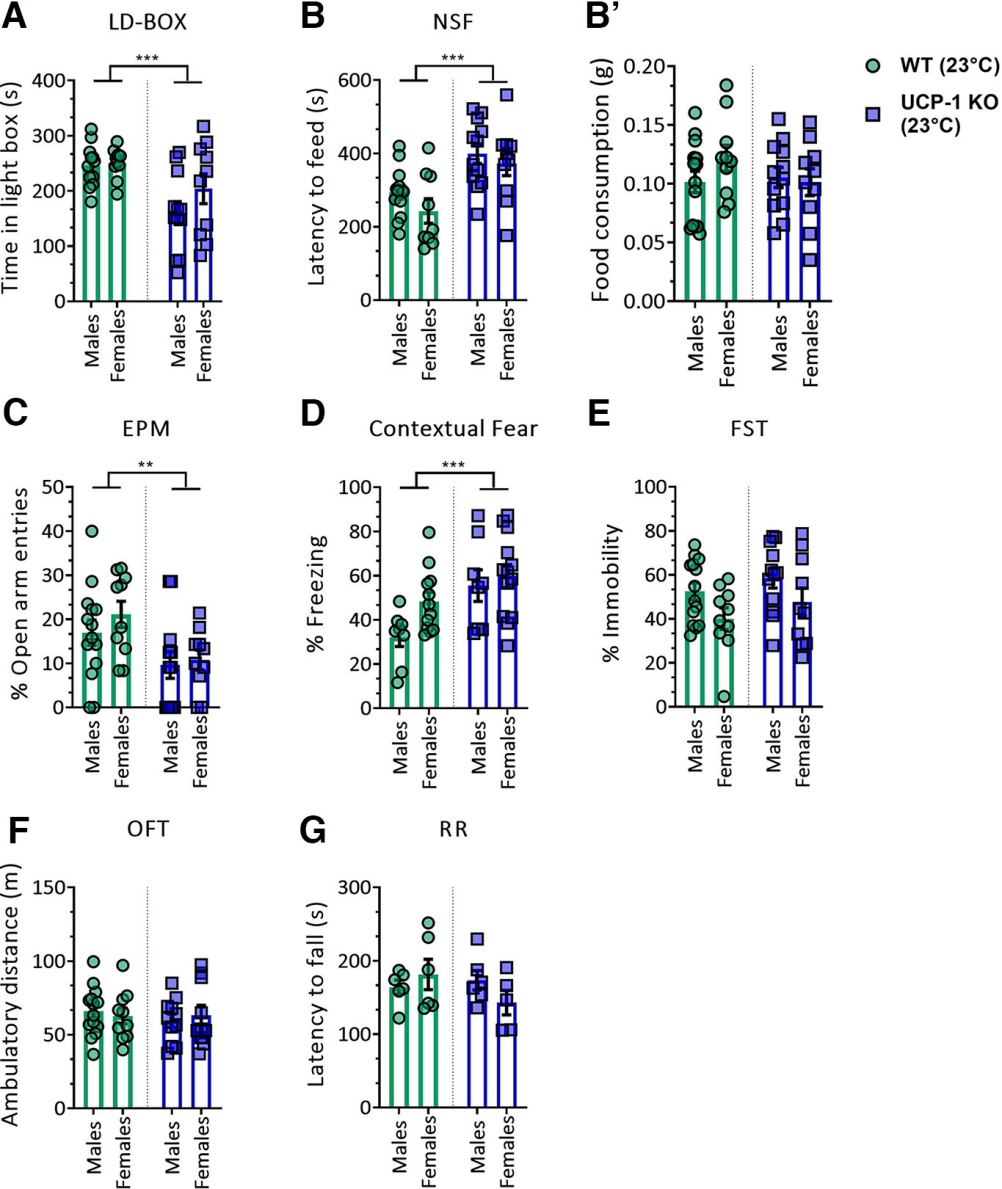
UCP-1 KO mice present with increased anxiety-related behavior at 23°C. ***A***, Time spent in the light compartment of the LD-BOX. ***B***, Latency to feed (in seconds) in the NSF test. ***B*′**, Home-cage food consumption (in grams) measured immediately after the NSF test. ***C***, Percentage of open arm entries in the EPM. ***D***, Percentage of freezing in the contextual fear test. ***E***, Percentage of immobility in the FST. ***F***, Total ambulatory distance traveled (in meters) in the OFT. ***G***, Latency to fall (in seconds) in the RR test. Data are presented as mean ± SEM. Data were analyzed by two-way ANOVA with genotype and sex as main factors: *N* = 5–14/group. Significant main genotype effects are indicated: *p < 0.05, **p < 0.01, ***p < 0.001. Absence of UCP-1 expression in UCP-1 KO and body weight of male and female UCP-1 KO and WT littermates are provided in Extended Data Figure 1-1.

We first conducted experiments at 23°C (regular housing temperature), corresponding to thermogenic conditions, which are highly dependent on UCP-1-mediated nonshivering thermogenesis (Feldmann et al., 2009). Female and male UCP-1 KO mice presented with enhanced innate anxiety-like behavior in the LD-BOX, the NSF, and the EPM. In the LD-BOX, UCP-1 KO mice spent significantly less time in the light compartment (Fig. 1*A*; *F*_(1,40)_ = 13.21, *p* = 0.0008); in the NSF test, latency to feed was enhanced in UCP-1 KO mice (Fig. 1*B*; *F*_(1,40)_ = 18.29, *p* = 0.0001). Home-cage food consumption immediately after the NSF was comparable between UCP-1 KO and WT littermates, of the test result, induced by changes in appetitive behavior (Fig. 1*B*′). Similarly, UCP-1 KO mice showed a decreased percentage of entries into the open arm in the EPM (Fig. 1*C*; *F*_(1,42)_ = 9.304, *p* = 0.0039), further confirming the results obtained in the LD-BOX and the NSF tests. The examination of learned fear responses in the fear-conditioning paradigm revealed augmented contextual fear responses in UCP-1 KO mice (Fig. 1*D*; *F*_(1,37)_ = 10.56, *p* = 0.0025).

10.1523/JNEUROSCI.2509-21.2022.f1-1 Figure 1-1 UCP-1 expression and body weight of UCP-1 KO and WT mice at 23°C. ***A***, UCP-1 expression in the hypothalamus of WT and UCP-1 KO mice. UCP-1 transcript levels were measured by qRT-PCR, and ΔCt values are presented. *N* = 4/group. ***B***, Body weight of WT and UCP-1 KO mice at regular housing temperature (23°C). Download Figure 1-1, TIF file.

There is a high degree of comorbidity between anxiety disorders and depressive disorders (Lamers et al., 2011). We therefore next explored depression-related behavioral despair in UCP-1 KO mice in the FST. No differences in immobility were detected between WT and UCP-1 KO in the FST (Fig. 1*E*). To further validate the behavioral results in the anxiety tests and exclude unspecific alterations in exploratory or motor activity and coordination as confounding factors, UCP-1 KO and WT littermates were tested in the OFT and the RR test. No effect of UCP-1 deficiency on distance traveled in the OFT (Fig. 1*F*) and the latency to fall off the rotating drum in the RR test (Fig. 1*G*) were found.

### Anxiety-like behavior is independent of UCP-1 at thermoneutrality

**Figure 2.**
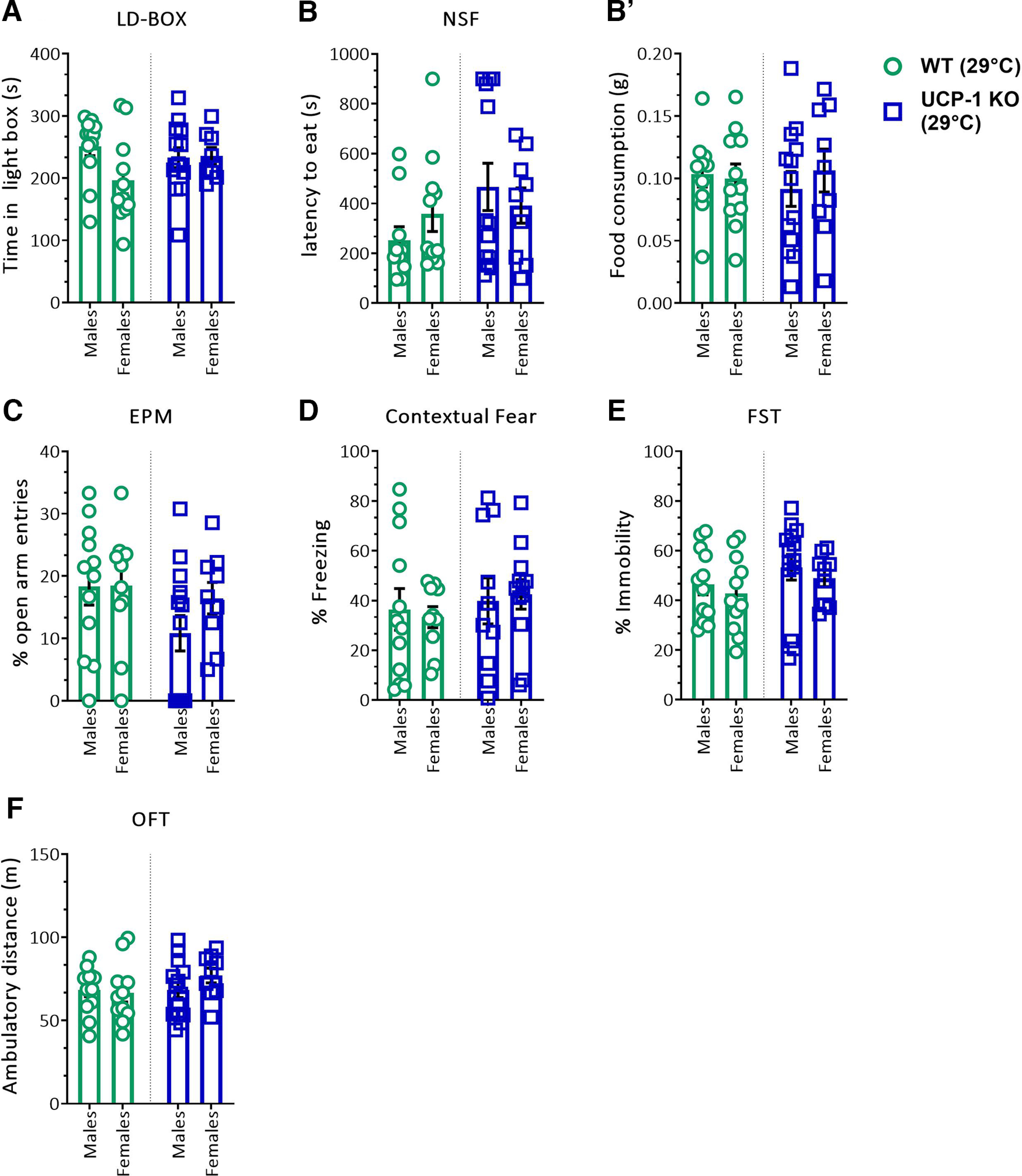
Anxiety-related behavior of UCP-1 KO mice at thermoneutrality (29°C) is unaltered. ***A***, Time spent in the light compartment of the LD-BOX. ***B***, Latency to feed (in seconds) in the NSF test. ***B*′**, Home-cage food consumption (in grams), measured immediately after the NSF test. ***C***, Percentage of open arm entries in the EPM. ***D***, Percentage of freezing in the contextual fear test. ***E***, Percentage of immobility in the FST. ***F***, Total ambulatory distance traveled (in meters) in the OFT. Data were analyzed by two-way ANOVA with genotype and sex as main factors; *n* = 8–15/group. Data are presented as mean ± SEM. Body weight of male and female UCP-1 KO and WT littermates are provided in Extended Data Figure 2-1.

Next, we asked whether the phenotype of UCP-1 KO mice was contingent on temperature conditions requiring thermogenesis. To address this question, we evaluated the emotional behavior of UCP-1 KO and WT mice at thermoneutrality (29°C), where the thermogenic requirement for BAT activation is minimal (Feldmann et al., 2009). A separate cohort of female and male UCP-1 WT and KO mice was housed at thermoneutrality directly after weaning and for at least 5 weeks before being subjected to the same battery of behavioral tests. The genotypes did not differ in any measures of innate anxiety in the LD-BOX (Fig. 2*A*), the NSF (Fig. 2*B*,*B*′), or the EPM (Fig. 2*C*). Furthermore, no differences in contextual fear (Fig. 2*D*) or in behavioral despair in the FST (Fig. 2*E*) were noted. Exploratory and locomotor activity in the OFT (Fig. 2*F*) remained unaltered in UCP-1 KO mice under thermoneutrality, as did the body weight of 3-month-old mice (Extended Data Fig. 2-1).

10.1523/JNEUROSCI.2509-21.2022.f2-1 Figure 2-1 Body weight of UCP-1 KO and WT mice at 29°C. Body weight of male and female UCP-1 KO and WT mice at thermoneutrality (29°C). *N* = 9–14/group. Data are presented as the mean ± SEM. Download Figure 2-1, TIF file.

These results demonstrate that UCP-1 is required for the regulation of anxiety-like behavior, but only under thermogenic conditions, suggesting an intricate relationship between the control of thermal and emotional homeostasis.

### Neural and endocrine stress mediators are not affected by UCP-1 deficiency

**Figure 3.**
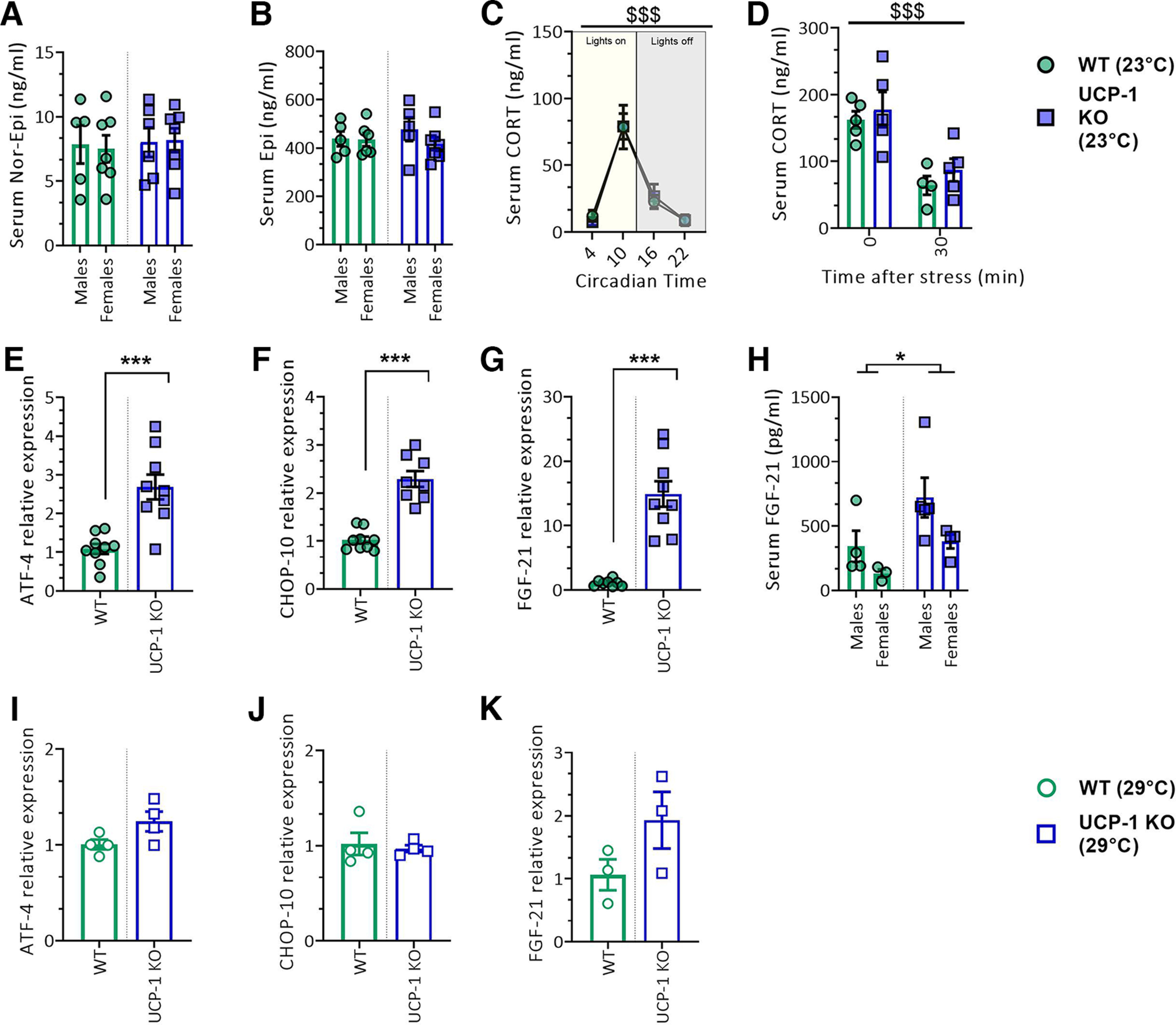
Serum catecholamines, corticosterone and integrated stress response markers in UCP-1 KO and WT mice. ***A***, ***B***, Serum nor-Epi levels (in nanograms per milliliter; ***A***) and serum Epi levels (in nanograms per milliliter; ***B***). Data were analyzed by two-way ANOVA with genotype and sex as main factors; *n* = 5–7/group. ***C***, Serum CORT levels (in nanograms per milliliter) in UCP-1 KO and WT mice at four different circadian time points. Data were analyzed by repeated-measures two-way ANOVA with genotype and circadian time point as main factors. Significant main effects of circadian time point effects are indicated: ^$$$^*p* < 0.001; *n* = 7–9/group. ***D***, Stress-induced CORT levels (in nanograms per milliliter) in UCP-1 KO and WT mice measured immediately or 30 min after the application of restraint stress. Data were analyzed by two-way ANOVA with genotype and time after stress as main factors. Significant main time after stress effects are indicated by ^$$$^*p* < 0.001; *n* = 4–5/group. ***E–G***, Relative expression of ATF-4 (***E***), CHOP-10 (***F***), and FGF-21 (***G***) in iBAT samples of UCP-1 KO mice and WT mice housed at regular housing temperature (23°C). Data were analyzed with Student's *t* test. *N* = 8–9/group. Significant differences are indicated as follows: *p < 0.05, **p < 0.01, ***p < 0.001. ***H***, Serum FGF-21 levels (in picograms per milliliter) in UCP-1 KO and WT mice housed at regular housing temperature (23°C). Data were analyzed by two-way ANOVA with genotype and sex as main factors; *n* = 3–5/group. Significant main genotype effects are indicated: *p < 0.05. ***I–K***, Relative expression of ATF-4 (***I***), CHOP-10 (***J***), and FGF-21 (***K***) in BAT samples of UCP-1 KO mice and WT mice housed at thermoneutrality (29°C). Data were analyzed with Student's *t* test. *N* = 3–4/group. Data are presented as mean ± SEM. A list with all primers sequences is provided in Extended Data Figure 3-1.

BAT is densely innervated by the sympathetic nervous system, whose activation stimulates UCP-1 activity (Cannon and Nedergaard, 2004) in response to cold exposure. It has been previously shown that circulating epinephrine (Epi) and nor-epinephrine (not-Epi) increase in response to cold exposure (Paakkonen and Leppaluoto, 2002). Against this background, and considering the important involvement of catecholamines in the regulation of emotions, specifically their relevance to fear and anxiety disorders (Alves et al., 2016; Martinho et al., 2020), we tested whether circulating levels of Epi and nor-Epi were differing between UCP-1 KO mice and WT controls. However, serum levels of both nor-Epi (Fig. 3*A*) and Epi (Fig. 3*B*) remained unaffected in UCP-1 KO mice. In light of the tight interactions between the autonomic nervous system and the hypothalamic-pituitary-adrenal (HPA) axis-mediated stress response, and taking into account the important contributions of glucocorticoids in the regulation of BAT activity (Ramage et al., 2016) and emotional function (Charmandari et al., 2005), we next examined the integrity of the humoral stress response system in UCP-1 KO mice. To this, serum CORT levels were assessed for their circadian rhythmicity and the sensitivity to acute stress exposure. At none of the four time points evaluated (CT4, CT10, CT16, and CT22) did CORT levels differ between UCP-1 KO and WT mice. As expected, corticosterone levels were highest just before the onset of the dark phase (CT10), regardless of the genotype (Fig. 3*C*; *F*_(3,56)_ = 31.16, *p* < 0.0001). Similarly, acute restraint stress-induced CORT levels were comparable between UCP-1 KO and WT littermates, both immediately, and 30 min after the termination of stress exposure (Fig. 3*D*). Stress-induced CORT levels were higher immediately after termination of the stress for both genotypes (Fig. 3*D*; *F*_(1,15)_ = 24.88, *p* < 0.001).

10.1523/JNEUROSCI.2509-21.2022.f3-1 Figure 3-1 Primer sequences. Download Figure 3-1, DOCX file.

Together these observations indicate that a derangement of either the neural or the humoral stress response system is unlikely to account for the increase in anxiety-like behavior in UCP-1 KO mice.

### Activation of the integrated stress response system in UCP-1 KO mice

Previous reports have shown that the effects of UCP-1 deficiency extend beyond thermoregulation and that UCP-1 ablation induces mitochondrial stress (Kazak et al., 2017) and integrated stress response (ISR) activation (Bond et al., 2018). Here, we quantified the expression of activating transcription factor 4 (ATF-4) and DNA damage-inducible transcript 3 (CHOP-10), two key players in the integrated stress response system (Wang et al., 2018), in the BAT of UCP-1 KO and WT littermates. We found that both ATF-4 (Fig. 3*E*; *p* = 0.0003) and CHOP-10 (Fig. 3*F*; *p* < 0.0001) transcripts were significantly upregulated in the BAT of UCP-1 KO mice housed at 23°C compared with WT littermates under the same temperature conditions. ATF-4 and CHOP-10 are key regulators of FGF-21, an integrated stress-responsive cytokine (Keipert et al., 2015) that is importantly involved in the control of energy homeostasis (Kharitonenkov et al., 2005). Quantification of FGF-21 expression revealed an increase in BAT FGF-21 levels in UCP-1 KO mice housed under thermogenic conditions (Fig. 3*G*; *p* < 0.0001) compared with WT littermates. At the same time, augmented levels of circulating FGF-21 were observed in UCP-1 KO (Fig. 3*H*; *F*_(1,12)_ = 6.567, *p* = 0.0249), as reported previously (Keipert et al., 2015). Results at thermoneutrality indicate that the changes in ATF-4, CHOP-10, and FGF-21 may be temperature dependent, as no significant differences in the expression of these transcripts were detected between genotypes at 29°C in the current dataset, while a trend was noted (Fig. 3*I–K*).

**Figure 4.**
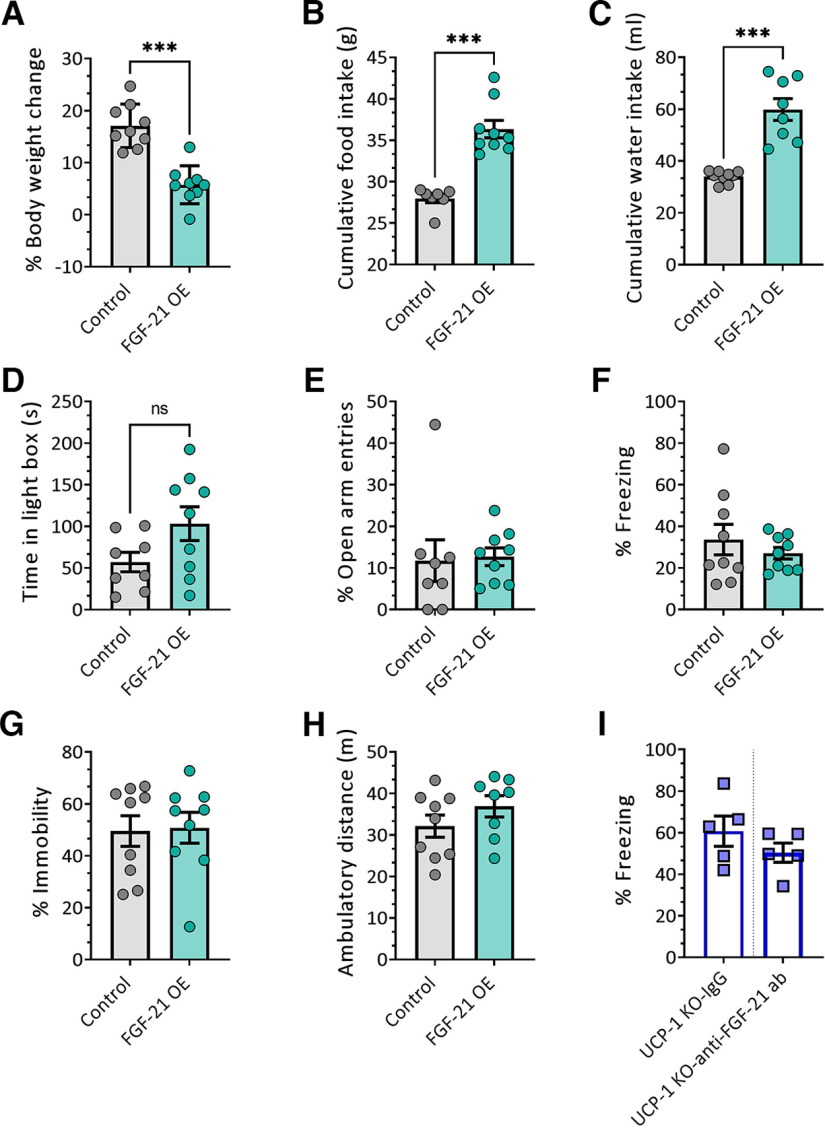
Systemic FGF-21 overexpression does not alter emotional behavior. ***A***, Percentage of body weight changes 3 weeks after viral overexpression of FGF-21. ***B***, ***C***, Cumulative food intake (***B***) and cumulative water intake (***C***) during the third week of viral overexpression of FGF-21. ***D***, Time spent in the light compartment of the LD-BOX; ***E***), percentage of open arm entries in the EPM (***F***), percentage of freezing in the contextual fear test (***G***), percentage of immobility in FST, and (***H***) ambulatory distance traveled (in meters) in the OFT in FGF-21-overexpressing and control mice. Data were analyzed with Student's *t* test. *N* = 7–9/group. *p* < 0.05, **p < 0.01, ***p < 0.001. ***I***, Percentage of immobility in the contextual fear test in UCP-1 KO mice treated with IgG or polyclonal anti-FGF-21 antibody. Data were analyzed with Student's *t* test. *N* = 5–9/group. *p < 0.05, **p < 0.01, ***p < 0.001. Data are presented as mean ± SEM. FGF-21 levels resulting from injection of AAV-FGF-21 are depicted in Extended Data Figure 4-1. The effects of FGF-21 overexpression in female mice are provided in Extended Data Figure 4-2.

Considering increasing evidence for a role of FGF-21 in brain function and behavior (Bookout et al., 2013), we next tested the possible mechanistic link between increased circulating levels of FGF-21 and the behavioral phenotype of UCP-1. To this end, we modeled augmented serum FGF-21 levels by systemic administration of an FGF-21-encoding AAV in male WT C57BL/6N mice. Injection in the tail vein with the FGF-21 encoding AAV induced high peripheral levels of FGF-21 in WT and significantly increased FGF-21 expression in the livers of injected mice (Extended Data Fig. 4-1). Mice overexpressing FGF-21 gained less weight than the control (empty AAV)-treated counterparts (Fig. 4*A*; *p* < 0.0001). Cumulative food (Fig. 4*B*; *p* < 0.0001) and water intake (Fig. 4*C*; *p* < 0.0001), measured during the third week of experiments, were significantly increased in in FGF-21-overexpressing mice, confirming previous reports that peripheral FGF-21 levels regulate food and water intake (Laeger et al., 2017; Turner et al., 2018).

10.1523/JNEUROSCI.2509-21.2022.f4-1 Figure 4-1 FGF-21 levels after injection of AAV-FGF-21. ***A***, Serum FGF-21 (in picograms per milliliter) levels 9 and 18 d after the injection of the FGF-21 encoding AAV. Data were analyzed by one-way ANOVA. *N* = 2/group. ***B***, Relative FGF-21 expression in the liver 18 d after the injection of the FGF-21 encoding AAV. Data were analyzed by Student's *t* test. *N* = 3/group. Data are presented as the mean ± SEM. Download Figure 4-1, TIF file.

10.1523/JNEUROSCI.2509-21.2022.f4-2 Figure 4-2 Physiological and behavioral consequences of FGF-21 overexpression in WT female mice. ***A***, Percentage of body weight changes 3 weeks after viral overexpression of FGF-21. ***B***, ***C***, Cumulative food intake (***B***) and cumulative water intake (***C***) during the third week of viral overexpression of FGF-21. ***D–H***, Time spent in the light compartment of the LD-BOX (***D***), the percentage of open arm entries in the EPM (***E***), the percentage of freezing in the contextual fear test (***F***), percentage of immobility in the FST (***G***), and ambulatory distance traveled (in meter) in the OFT (***H***) in FGF-21 overexpressing and control female mice. Data were analyzed with Student's *t* test. *N* = 6–10/group. *p* < 0.05, **p < 0.01, ***p < 0.001. Data are presented as the mean ± SEM. Download Figure 4-2, TIF file.

However, FGF-21 overexpression had no effects on either anxiety-related behavior, as seen in LD-BOX, EPM, and fear-conditioning tests, or depression-related behavior (Fig. 4*D–G*). In addition, FGF-21 overexpression did not modulate locomotion in the OFT (Fig. 4*H*). These results indicate that increasing peripheral FGF-21 levels is not sufficient to mimic the behavioral phenotype of UCP-1 KO mice. To exclude sex-specific effects of FGF-21 overexpression, the experiments were repeated in a cohort of female WT C57BL/6N mice (Extended Data Fig. 4-2). In contrast to male mice, FGF-21 overexpression in female mice did not result in reduced body weight (Extended Data Fig. 4-2*A*). However, both food and water intake measured during the third week of experiments were significantly increased in response to heightened FGF-21 levels (Extended Data Fig. 4-2*B*,*C*). Also in female WT mice, increased peripheral FGF-21 levels did not alter emotional behavior or locomotor activity (Extended Data Fig. 4-2*D–H*).

We then asked whether FGF-21 was required for the anxiogenic phenotype of UCP-1 KO mice. We tested this contingency using a polyclonal anti-FGF-21 antibody to block its activity before behavioral testing (Liu et al., 2019; Li et al., 2020). Yet, no effect of FGF-21 inhibition on contextual fear in UCP-1 KO animals was observed (Fig. 4*I*). Jointly, our results demonstrate that increased levels of FGF-21 are not mechanistically related to the behavioral disturbances displayed by UCP-1 KO mice.

### The behavioral phenotype of UCP-1 KO mice persists after surgical removal of BAT

**Figure 5.**
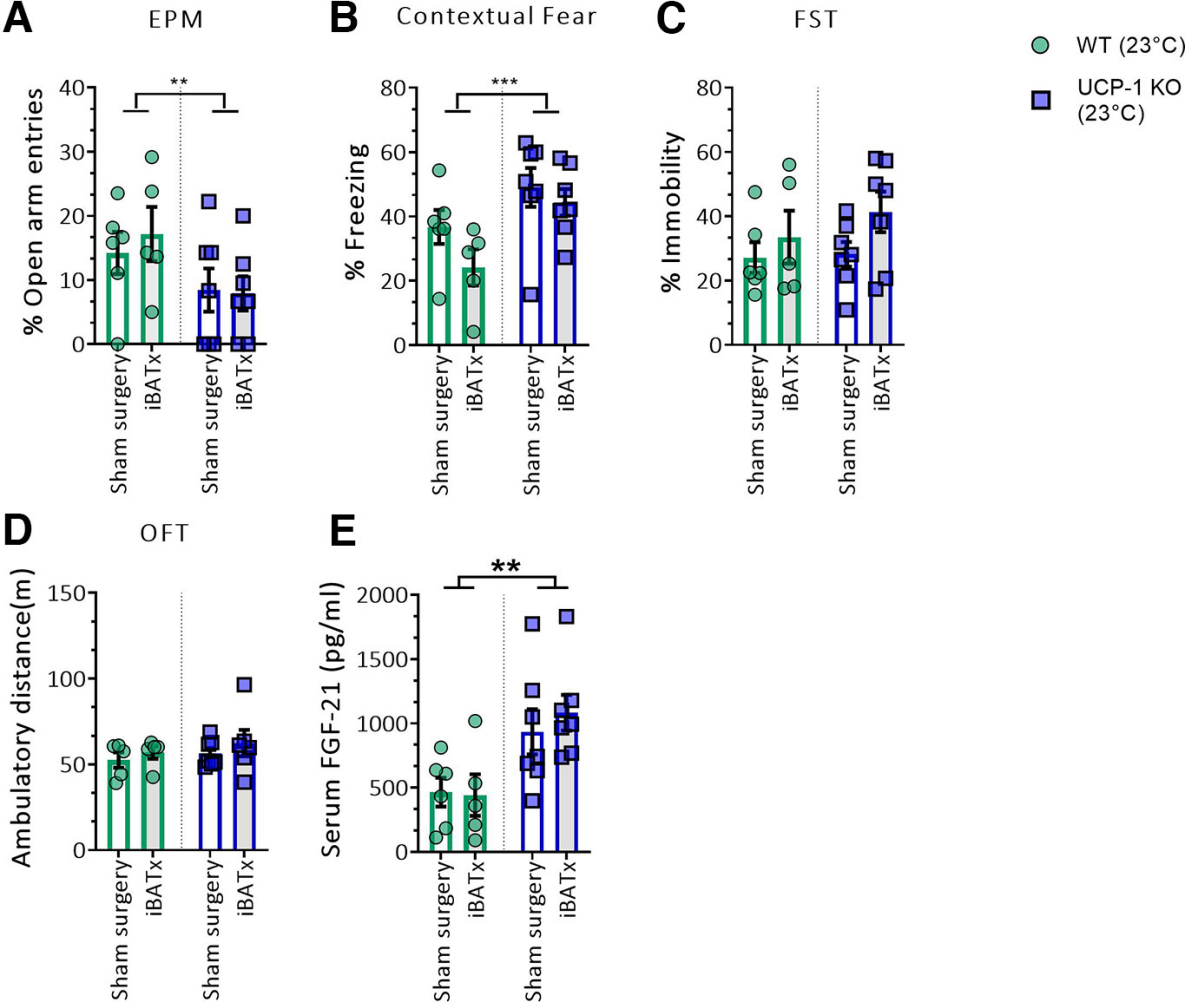
iBATx does not alter emotional behavior. ***A–E***, Percentage of open arm entries in the EPM (***A***), percentage of immobility in the contextual fear test (***B***), percentage of immobility in FST (***C***), ambulatory distance traveled (in meters) in the OFT (***D***), and serum FGF-21 levels (in picograms per milliliter) in UCP-1 KO and WT mice after iBATx or sham surgery (***E***), respectively. Data were analyzed by two-way ANOVA with genotype and BAT surgery (iBATx vs sham) as main factors; *n* = 5–7/group. Significant main effects of genotype are indicated: *p* < 0.05, **p < 0.01, ***p < 0.001. Data are presented as mean ± SEM. The experimental design of the iBATx experimented is represented in Extended Data Figure 5-1.

BAT–brain communication can be mediated through humoral signals or neural afferents. To further test whether BAT adipokines, other than FGF-21, or neural afferents from BAT to brain (Ryu et al., 2015) could contribute to the behavioral consequences of UCP-1 deficiency, interscapular BAT (iBAT) was surgically removed (iBATx) from adult UCP-1 KO and control mice (WT). A brief overview of the four experimental groups is provided in Extended Data Figure 5-1*A* [sham-operated UCP-1 KO mice (KO-Sham)] or iBAT removal surgery (KO-iBATx) and WT mice with either sham surgery (WT-Sham) or iBAT removal (WT-iBATx). The behavioral effects of iBATx were evaluated 4 weeks after surgery, when no evidence for iBAT regeneration was observed (Extended Data Fig. 5-1*A*,*B*). The previously noted genotype-dependent behavioral performance in innate and learned fear (i.e., EPM and contextual fear) was preserved also in iBATx groups, indicating that BAT surgical removal did not affect anxiety-like behavior in either UCP-1 KO or WT mice (Fig. 5*A*: *F*_(1,21)_ = 5.021, *p* = 0.0360; Fig. 5*B*: *F*_(1,21)_ = 9.154, *p* = 0.0064). iBATx did also not alter behavioral despair in the FST or exploratory locomotor activity in the OFT, in either UCP-1 KO or WT mice (Fig. 5*C*,*D*). iBATx also had no effect on FGF-21 levels in either UCP-1 KO or WT mice, as the previously found genotype-dependent effect was confirmed (Fig. 5*E*; *F*_(1,21)_ = 13.18, *p* = 0.0016), with UCP-1 KO mice presenting higher circulating FGF-21 levels than WT controls, regardless of the surgical treatment.

10.1523/JNEUROSCI.2509-21.2022.f5-1 Figure 5-1 Design of iBATx experiments. ***A***, Overview of the experimental groups included in iBATx experiments. ***B***, No signs of iBAT regeneration were observed 4 weeks after iBAT surgical removal. Download Figure 5-1, TIF file.

Together, our results show that the increase in anxiety observed in UCP-1 KO mice is not induced by enhanced levels of FGF-21 or other direct humoral or neural communicatory signals from BAT to brain, proposing a role for brain-expressed UCP-1 in the regulation of emotional behavior.

## Discussion

Clinical evidence strongly supports a bidirectional association between emotional and metabolic disturbances. Both pathologies are of high prevalence and significant socioeconomic relevance (Wittchen et al., 2011). Yet, our understanding about the mechanistic basis mediating the comorbidity between affective and metabolic disorders is very limited. This is surprising, especially considering that current treatment options remain unsatisfactory for a high number of patients with anxiety and depressive disorders (Craske et al., 2017) and gaining insight into novel aspects about the underlying pathophysiological principles has the potential for opening up new avenues for alternative therapeutic interventions.

Here we set out to explore the role of UCP-1, a key metabolic regulator known for its essential function in BAT-dependent non-shivering thermogenesis, in emotional behavior in mice. We find that the depletion of UCP-1 in a genetic mouse model is associated with an anxiogenic behavioral phenotype that is manifested only under thermogenic conditions. Although the systemic endocrine and neural stress system is unaffected, UCP-1 KO mice show elevated ISR markers and related enhanced levels of FGF-21. However, increasing systemic FGF-21 levels in WT mice did not phenocopy augmented anxiety-like behavior observed in UCP-1 KO mice, and blocking FGF-21 activity in KO mice did not rescue their phenotype. Surgical iBAT removal had no effect on either the increased anxiety-like behavior or the elevated FGF-21 levels in UCP-1 KO mice. Thus, we observe a temperature-dependent regulation of anxiety-like behavior by UCP-1, which is not contingent on BAT.

The hypothesis that UCP-1 could serve as a molecular link between metabolic and emotional disorders, was originally motivated by the consideration that (1) UCP-1 is strongly expressed in BAT, and BAT is critical to energy control and consequently relevant to metabolic dysfunctions, including those associated with affective disorders (e.g., obesity and type II diabetes mellitus); and (2) A bidirectional communication between BAT and the brain exists, using both neural and endocrine pathways (Ryu et al., 2015; Villarroya et al., 2017).

Indeed, we find the consequences of UCP-1 deficiency to be reflected in a highly specific behavioral phenotype with robust and selective increase in innate and learned fear responses, exclusively under thermogenic conditions. There are different possibilities to explain this interaction between UCP-1 and temperature on emotional regulation: (1) under thermogenic conditions UCP-1-deficient mice compensate for the diminished BAT activity through the recruitment of alternative methods of thermogenesis, which also affect brain function and behavior; (2) in a thermogenic temperature environment, UCP-1-deficient BAT releases adipokines that also act on the brain; and (3) UCP-1 has direct effects in the brain where its activity/function is also temperature dependent.

We tested the possible recruitment of compensatory thermogenic mechanisms focusing first on involvement of the catecholamines nor-epinephrine and epinephrine, which are increased in response to cold exposure because of sympathetic nervous system activation and can modulate core body temperature and anxiety (Alves et al., 2016; Martinho et al., 2020). However, serum levels of nor-epinephrine and epinephrine were unaltered in UCP-1 KO mice. Along the same lines, we examined the integrity of the HPA axis, being central to metabolic and emotional control (Charmandari et al., 2005; Moraitis et al., 2017), but we found no evidence for dysfunctional circadian regulation or stress-induced release of corticosterone in UCP-1 KO mice.

We then explored the possible differential release of batokines from the UCP-1-deficient BAT focussing on those with impact on the brain. Indeed, in agreement with earlier reports (Keipert et al., 2015), we observed significantly elevated BAT and serum levels of FGF-21 in UCP-1 KO mice, possibly resulting from enhanced ISR reflected in increased expression of BAT ATF-4 and CHOP-10. FGF-21 was a prime candidate for further investigation as a possible mediator between BAT and brain function as it is able to cross the blood–brain barrier (Hsuchou et al., 2007), has been shown to centrally (Owen et al., 2014) modulate important physiological functions (including water, alcohol, and sugar intake; Talukdar et al., 2016; Søberg et al., 2018; Turner et al., 2018), and binds at key regions in the brain controlling emotional behavior, such as the hypothalamus (Bookout et al., 2013; Owen et al., 2014). However, we found that FGF-21 was neither sufficient nor required for the anxiogenic phenotype observed in UCP-1 KO mice, as neither FGF-21 overexpression in WT animals nor blocking of FGF-21 activity in KO mice impacted the fear response.

Since FGF-21 is not the only hormone released from BAT (Kiefer, 2017; Villarroya et al., 2017), and BAT–brain communication can be enabled by neural afferents from BAT to key regions of the brain (Ryu et al., 2015), we surgically removed iBAT, the largest brown adipose depot in mice (Ikeda et al., 2018), from a cohort of WT and UCP-1 KO mice. A similar approach had been previously used to identify that iBAT in mice has the capacity to secrete and modulate circulating interleukin-6 levels in response to stress (Qing et al., 2020). Yet, surgical removal of iBAT neither ameliorated the behavioral abnormalities of UCP-1 KO mice nor affected the behavior of WT counterparts, suggesting that the behavioral consequences of UCP-1 deficiency are independent of its function in the BAT. Interestingly, iBATx also did not normalize the heightened FGF-21 levels observed in UCP-1 KO mice. Previous studies have suggested BAT as the major driver of increased FGF-21 levels in UCP-1 KO housed under conditions of thermal stress (Keipert et al., 2015). The results obtained herein indicate that the removal of iBAT is not sufficient to restore FGF-21 levels and that other fat depots, such as subcutaneous adipose tissue and organs, including liver and pancreas (Fon Tacer et al., 2010; Markan et al., 2014), may contribute to the heightened FGF-21 levels observed in UCP-1 KO mice.

Using the heightened anxiety response of UCP-1 KO mice in the contextual fear paradigm as a proxy of their emotional phenotype allows integration of the observed effects across models and paradigms in the present study. Jointly, these results indicate that neither peripheral responses to thermogenic conditions nor direct humoral or neural communicatory signals from BAT to brain are relevant to the behavioral phenotype of UCP-1 KO mice, which suggests a direct role for brain-expressed UCP-1 in the regulation of emotional behavior. With regard to brain function, UCP-1 has hitherto only been associated with sleep regulation (Szentirmai and Kapás, 2014), and a central effect of UCP-1 in the regulation of energy balance through the control of food intake has been proposed (Okamatsu-Ogura et al., 2011). While previously the presence of other members of the UCP family of proteins in the brain has been affirmed and a role in neuronal function been demonstrated (for review, see Andrews et al., 2005), UCP-1 expression in the mouse brain (Lengacher et al., 2004; Wang et al., 2019) has remained contradictory. However, a recent study using UCP1-cre reporter mice convincingly delineated the active expression of UCP1 in the mouse brain, with abundant levels of UCP-1 in the VMH and the amygdala (Claflin et al., 2022). Indeed, in the present study we also confirmed UCP-1 expression in the hypothalamus of WT mice.

Relevant to the anxiogenic phenotype of UCP-1 KO mice, VMH is a core structure of the innate defense network of the brain (Dielenberg and McGregor, 2001; Cheung et al., 2015) and receives direct input from neurons of the amygdala (Yamamoto et al., 2018). Importantly, amygdala neurons project to glutamatergic neurons in the VMH (Yamamoto et al., 2018), where UCP-1-expressing neurons are exclusively glutamatergic (Claflin et al., 2022). Thus, it can be hypothesized that UCP-1 may constitute a hitherto unknown molecular mediator of the innate defense network to contribute to the control of anxiety-like behavior. Important follow-up experiments to further investigate the central role of UCP-1 will like rely on examining the consequences of brain/nucleus-specific UCP-1 knockdown.

In summary, we here reveal a role for UCP-1 in the temperature-dependent regulation of anxiety-like behavior and propose this function to be mediated through a central effect of UCP-1 in brain regions forming part of the innate defense networks, suggesting UCP-1 as molecular link between metabolic and anxiety disorders.
