# Supplementary figures and images for: Uncoupling Protein-1 Modulates Anxiety-Like Behavior in a Temperature-Dependent Manner
Source: J Neurosci. 2022 Oct 5;42(40):7659–72. doi: 10.1523/JNEUROSCI.2509-21.2022 (PMC9546448; doi:10.1523/JNEUROSCI.2509-21.2022)

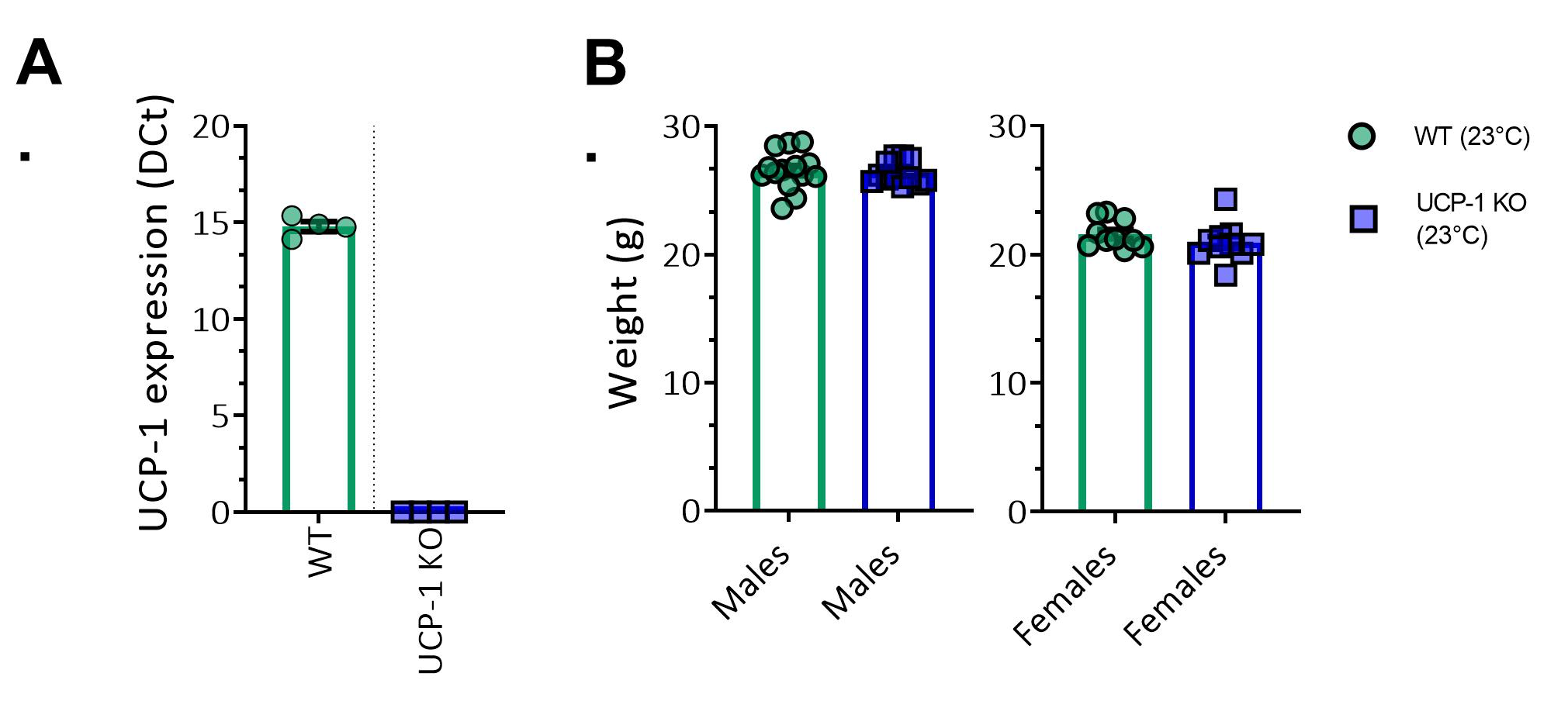

Supplement: Figure 1-1 — UCP-1 expression and body weight of UCP-1 KO and WT mice at 23°C. A, UCP-1 expression in the hypothalamus of WT and UCP-1 KO mice. UCP-1 transcript levels were measured by qRT-PCR, and ΔCt values are presented. N = 4/group. B, Body weight of WT and UCP-1 KO mice at regular housing temperature (23°C). Download Figure 1-1, TIF file. [file ns-JN-RM-2509-21-s01.tif]

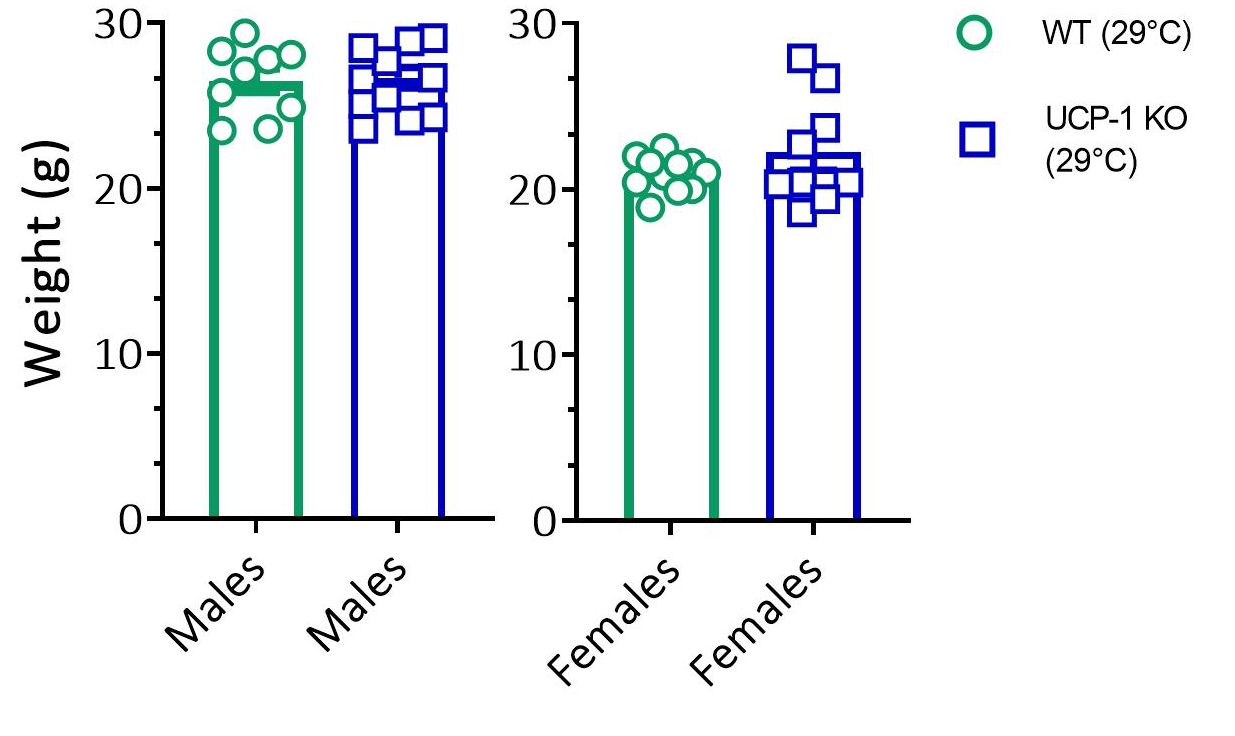

Supplement: Figure 2-1 — Body weight of UCP-1 KO and WT mice at 29°C. Body weight of male and female UCP-1 KO and WT mice at thermoneutrality (29°C). N = 9–14/group. Data are presented as the mean ± SEM. Download Figure 2-1, TIF file. [file ns-JN-RM-2509-21-s02.tif]

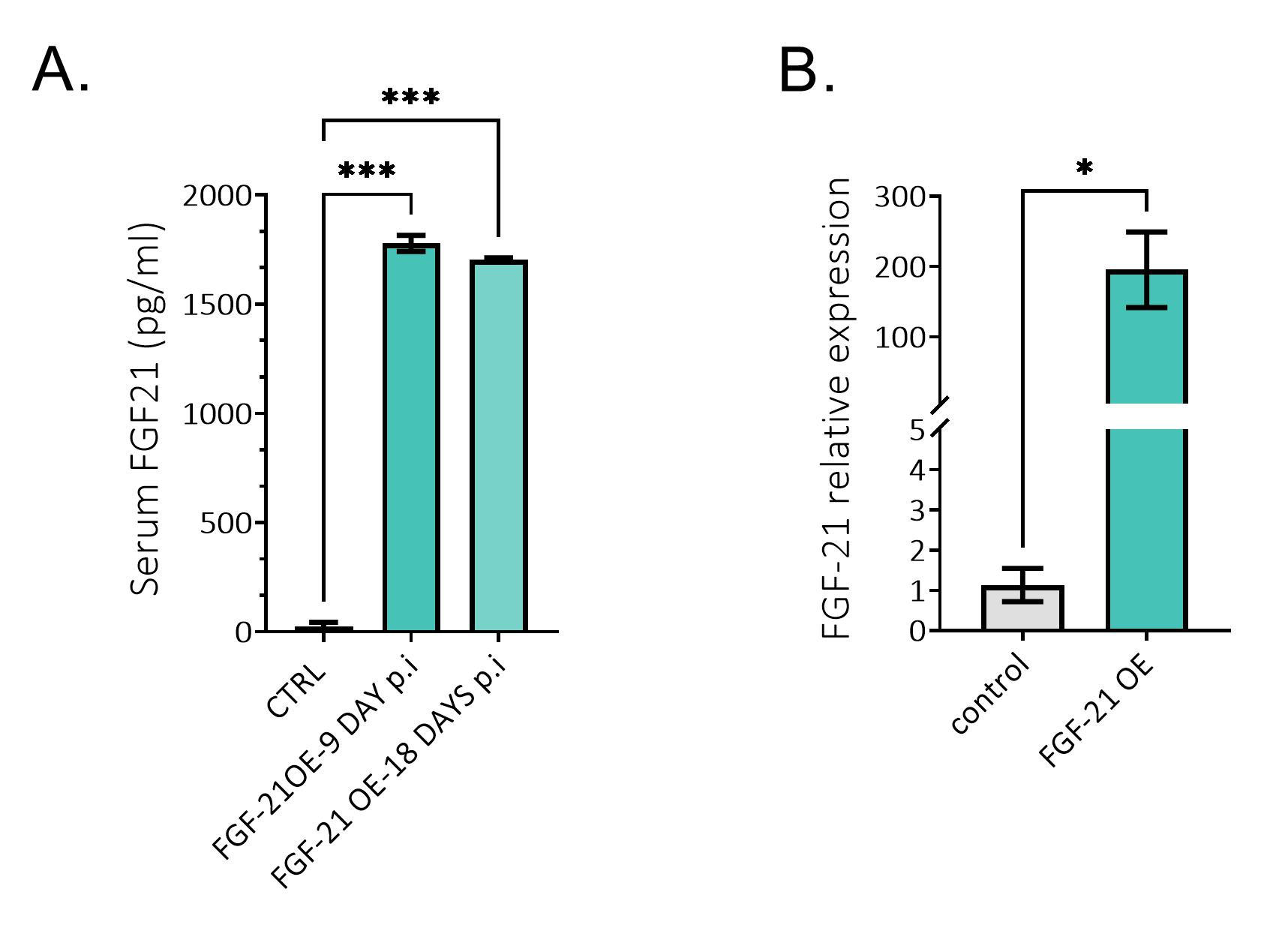

Supplement: Figure 4-1 — FGF-21 levels after injection of AAV-FGF-21. A, Serum FGF-21 (in picograms per milliliter) levels 9 and 18 d after the injection of the FGF-21 encoding AAV. Data were analyzed by one-way ANOVA. N = 2/group. B, Relative FGF-21 expression in the liver 18 d after the injection of the FGF-21 encoding AAV. Data were analyzed by Student's t test. N = 3/group. Data are presented as the mean ± SEM. Download Figure 4-1, TIF file. [file ns-JN-RM-2509-21-s03.tif]

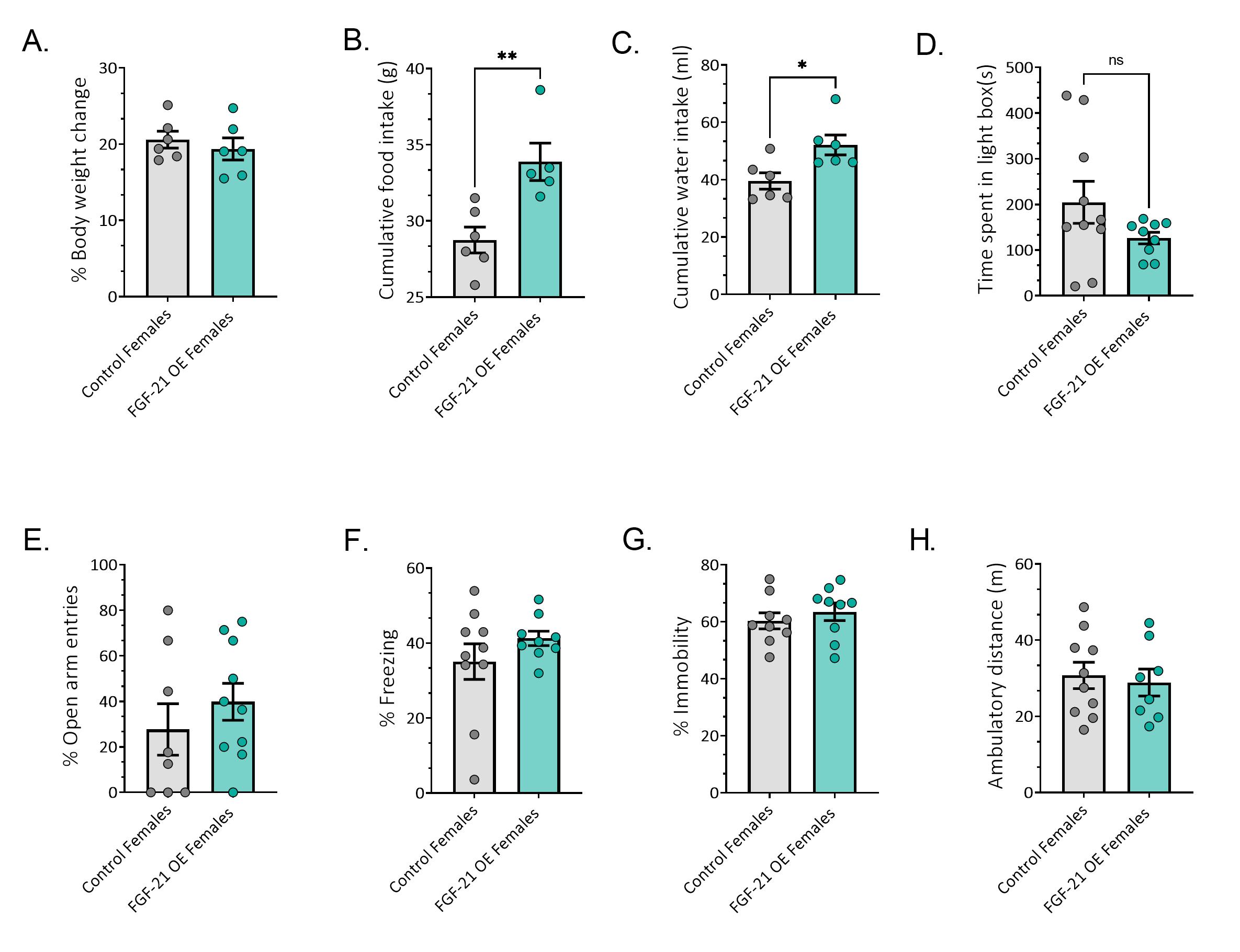

Supplement: Figure 4-2 — Physiological and behavioral consequences of FGF-21 overexpression in WT female mice. A, Percentage of body weight changes 3 weeks after viral overexpression of FGF-21. B, C, Cumulative food intake (B) and cumulative water intake (C) during the third week of viral overexpression of FGF-21. D–H, Time spent in the light compartment of the LD-BOX (D), the percentage of open arm entries in the EPM (E), the percentage of freezing in the contextual fear test (F), percentage of immobility in the FST (G), and ambulatory distance traveled (in meter) in the OFT (H) in FGF-21 overexpressing and control female mice. Data were analyzed with Student's t test. N = 6–10/group. p < 0.05, **p < 0.01, ***p < 0.001. Data are presented as the mean ± SEM. Download Figure 4-2, TIF file. [file ns-JN-RM-2509-21-s04.tif]

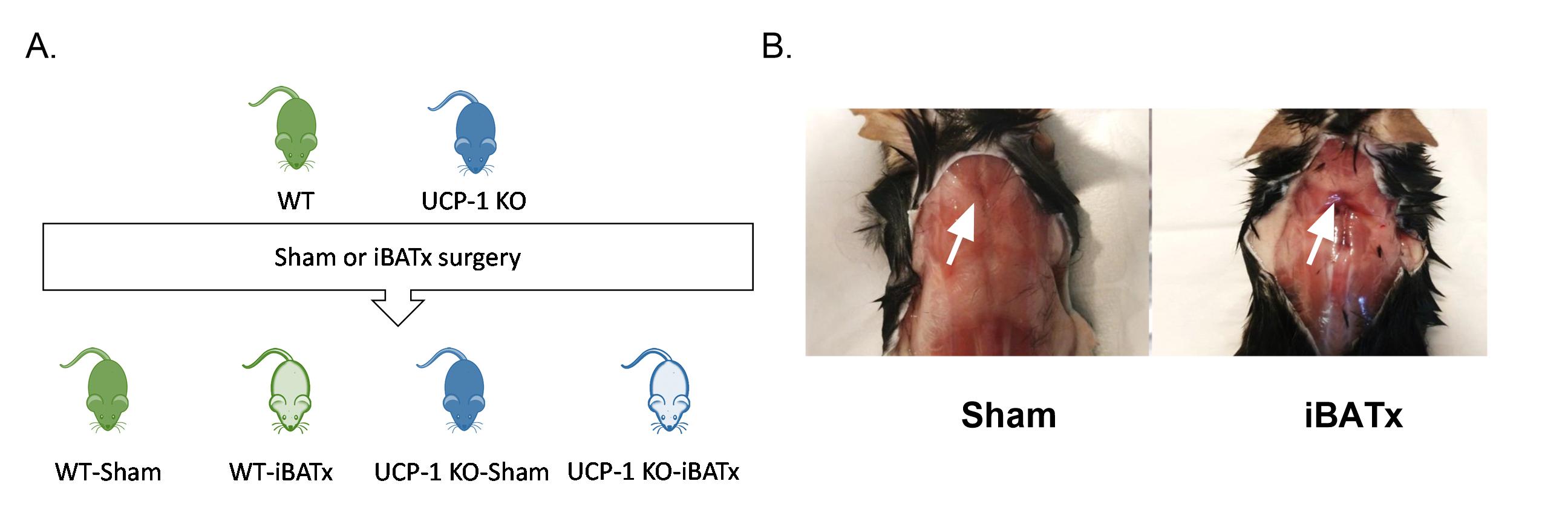

Supplement: Figure 5-1 — Design of iBATx experiments. A, Overview of the experimental groups included in iBATx experiments. B, No signs of iBAT regeneration were observed 4 weeks after iBAT surgical removal. Download Figure 5-1, TIF file. [file ns-JN-RM-2509-21-s05.tif]
