# Supplementary material for: Uncoupling Protein-1 Modulates Anxiety-Like Behavior in a Temperature-Dependent Manner
Source: J Neurosci. 2022 Oct 5;42(40):7659–72. doi: 10.1523/JNEUROSCI.2509-21.2022 (PMC9546448; doi:10.1523/JNEUROSCI.2509-21.2022)
Supplement: Figure 3-1 — Primer sequences. Download Figure 3-1, DOCX file. [file ns-JN-RM-2509-21-s06.docx]

**Figure 3-1. Primer sequences**

| Beta-actin forward | ATGGTGGGAATGGGTCAGAAG |
| --- | --- |
| Beta-actin reverse | TCTCCATGTCGTCCCAGTTG |
| 36B4 forward | TCTCCATGTCGTCCCAGTTG |
| 36B4 reverse | TCTCCATGTCGTCCCAGTTG |
| FGF21 forward | TCTCCATGTCGTCCCAGTTG |
| FGF21 reverse | TCTCCATGTCGTCCCAGTTG |
| ATF-4 forward | CCTGAACAGCGAAGTGTTGG |
| ATF-4 reverse | TGGAGAACCCATGAGGTTTCAA |
| CHOP-10 forward | AAGCCTGGTATGAGGATCTGC |
| CHOP-10 reverse | TTCCTGGGGATGAGATATAGGTG |
| UCP-1 forward | TCAGGATTGGCCTCTACGAC |
| UCP-1 reverse | TGCATTCTGACCTTCACGAC |
